# The Role of a Longitudinal, Multidisciplinary Clinic in Building a Unique Research Collaborative

**DOI:** 10.3389/fonc.2022.857699

**Published:** 2022-04-08

**Authors:** Alexandria A. Gonzales, Alexander Mastrolonardo, Kenna Winget, Malavan Ragulojan, Adam J. Fleming, Sheila K. Singh

**Affiliations:** ^1^ Pediatric Brain Tumour Study Group, McMaster University, Hamilton, ON, Canada; ^2^ School of Interdisciplinary Science, McMaster University, Hamilton, ON, Canada; ^3^ Michael G. DeGroote School of Medicine, McMaster University, Hamilton, ON, Canada; ^4^ School of Kinesiology and Health Studies, Queen’s University, Kingston, ON, Canada; ^5^ Division of Hematology and Oncology, Department of Pediatrics, McMaster University, Hamilton, ON, Canada; ^6^ Division of Neurosurgery, Department of Surgery, McMaster University, Hamilton, ON, Canada

**Keywords:** neuro-oncology, teamwork, research collaboration, multidisciplinary clinic, team dynamics, research team, multidisciplinary collaboration, multigenerational teams

## Abstract

Multidisciplinary neuro-oncology clinics allow collaboration between various specialties and training levels. Building a tenable clinical research program based in the longitudinal dialogue and practice of collaborative clinicians and trainees can bridge clinical observations to research execution. However, forming a research team around a multidisciplinary clinic’s activities is constrained by a lack of literature or guidelines. As well, challenges in sustaining team logistics, communication, and productivity can persist without a standardized team framework. This perspective discusses the state of research teams in clinical oncology, and uses experiences from the McMaster Pediatric Brain Tumour Study Group to guide those seeking to form a research team based on the collective activities and observations of a multidisciplinary clinic.

## Introduction

Multidisciplinary research teams consist of many individuals with differing skills and knowledge. With a variety of expertise, these teams produce novel and robust research. To be successful, it is crucial for research teams to develop strategies and define goals (1). Currently, research teams in pediatric hematology/oncology nursing and geriatric oncology have documented lessons from their success (2, 3). However, a paucity of literature exists in the context of pediatric neuro-oncology, and how clinic activities can be leveraged to guide an adjacent research team. Recommendations on translating clinical observations to research endeavours and establishing a sustainable research team can ensure success and longevity of these teams within the field. These recommendations also form an initial framework that can eventually mould into a standardized framework among research teams in oncology and other specialties.

The McMaster Pediatric Brain Tumour Study Group (PBTSG) was founded in 2015, originating from the abundance of unanswered questions raised at the weekly pediatric neuro-oncology multidisciplinary case conferences (MCC) at McMaster Children’s Hospital in Ontario, Canada. This multidisciplinary research collaborative represents a clinical research network of over 20 allied health professionals and 15 students who have developed a comprehensive brain tumor patient database that has been mined for clinical research questions, generated multiple publications and presentations, and serves as a clinically annotated repository for future studies. The PBTSG found its roots in the thought-provoking discussions that emerged at the multidisciplinary longitudinal pediatric neuro-oncology clinic. Over the past century, the clinic has fostered extended case discussions of patients longitudinally followed for their lives after being treated for a childhood central nervous system tumour. The clinic is comprised of specialists from diverse backgrounds, including pediatric neurosurgery, neuro-oncology, nursing and neuro-radiology; radiation oncology, neuro-psychology, occupational and physiotherapy, child life, social work, exercise medicine, nutrition and endocrinology.

Although the multidisciplinary format of the MCC facilitated great discussion, one of the leading pediatric neurosurgeons recognized that clinicians sometimes lack protected research time to translate these conversations into research. Formal research often requires complex coordination, as well as time for complex analyses and creativity in research design. For these reasons, it is suggested that multidisciplinary teams are optimal for sharing leadership and responsibility among team members (4). As the clinicians were receiving many requests from learners to engage in research projects, it was these individuals who could collaborate with the clinicians while mitigating time constraints. Ranging from undergraduate university students to clinical oncology fellows, these bright and energetic learners could seek training and mentorship from whichever multidisciplinary faculty member was most expert in the line of inquiry. The director of the pediatric neuro-oncology clinic became a logical co-director of the group, with their specialized practice and content expertise. Following the addition of a biostatistician and the clinical epidemiological and study design expertise of their team, the PBTSG was born.

Over the past 6 years (and even through the COVID-19 pandemic) the PBTSG has generated over 30 research publications and conference presentations, with an equal number in development. Our group has sustained its research output and growth by developing a logistical foundation and cohesive team structure. We will discuss the framework of the PBTSG, reflect on challenges and strategies involved in sustaining a multidisciplinary team, and provide recommendations to oncology clinics seeking to form similar research groups.

## Challenges and Strategies

### Translating Clinical Observations Into Research Data

Bridging observations from clinic activities into research endeavours requires ethics clearance for patient data collection. Given the necessity of maintaining patient confidentiality, the progress of clinical projects requires significant time allotted for research ethics board review. Moreover, the risk of storing patient data across unsecure platforms poses the challenge of balancing practical data access with data security.

A research ethics board application for a universal clinical database was a starting point for commencing multiple research projects more efficiently. Research Electronic Data Capture (REDCap) was the software chosen to store the data, as it warrants convenience without compromising health law compliance and security (5). As well, uniting the entire group under a single ethics application enables us to provide yearly updates that maintain concordance with evolving data privacy regulations.

Once new members complete privacy and ethics training provided by the research ethics board, they are oriented into the PBTSG through our online curriculum. Curated by existing PBTSG members, the aim of the curriculum is to provide new members with essential knowledge in pediatric brain tumours and database entry. New members are assigned broad literature on pediatric brain tumours. Following this, they are trained in database entry through interactive modules. These original modules teach necessary skills in data navigation, acquisition, and transfer from our institution’s electronic medical record system to the REDCap software. In developing these skills, pertinent health information can be routinely translated into data for our studies. The curriculum does not contain any confidential information, and is housed on our encrypted website. Together, these promote easy accessibility and dissemination of the curriculum to both our members and those curious about neuro-oncology.

Through populating the database and working as a team, members organically develop their own research questions, which they cross-reference *via* literature searches to ensure their novelty. Once a novel question is determined and vetted by the group, appropriate expert faculty mentors are assigned and study design, data analysis, and manuscript and presentation preparation ensue. Undergraduate students may use their group participation and projects in the form of an undergraduate thesis course, with every student aiming to prepare a manuscript for publication. To date, the productivity of the PBTSG has been promising, addressing complex and diverse research topics such as discerning the neuroradiological correlates of cerebellar mutism (6), and eliciting the proximate and ultimate determinants of quality of life in pediatric brain tumour patients (manuscript in preparation).

**Figure 1:**
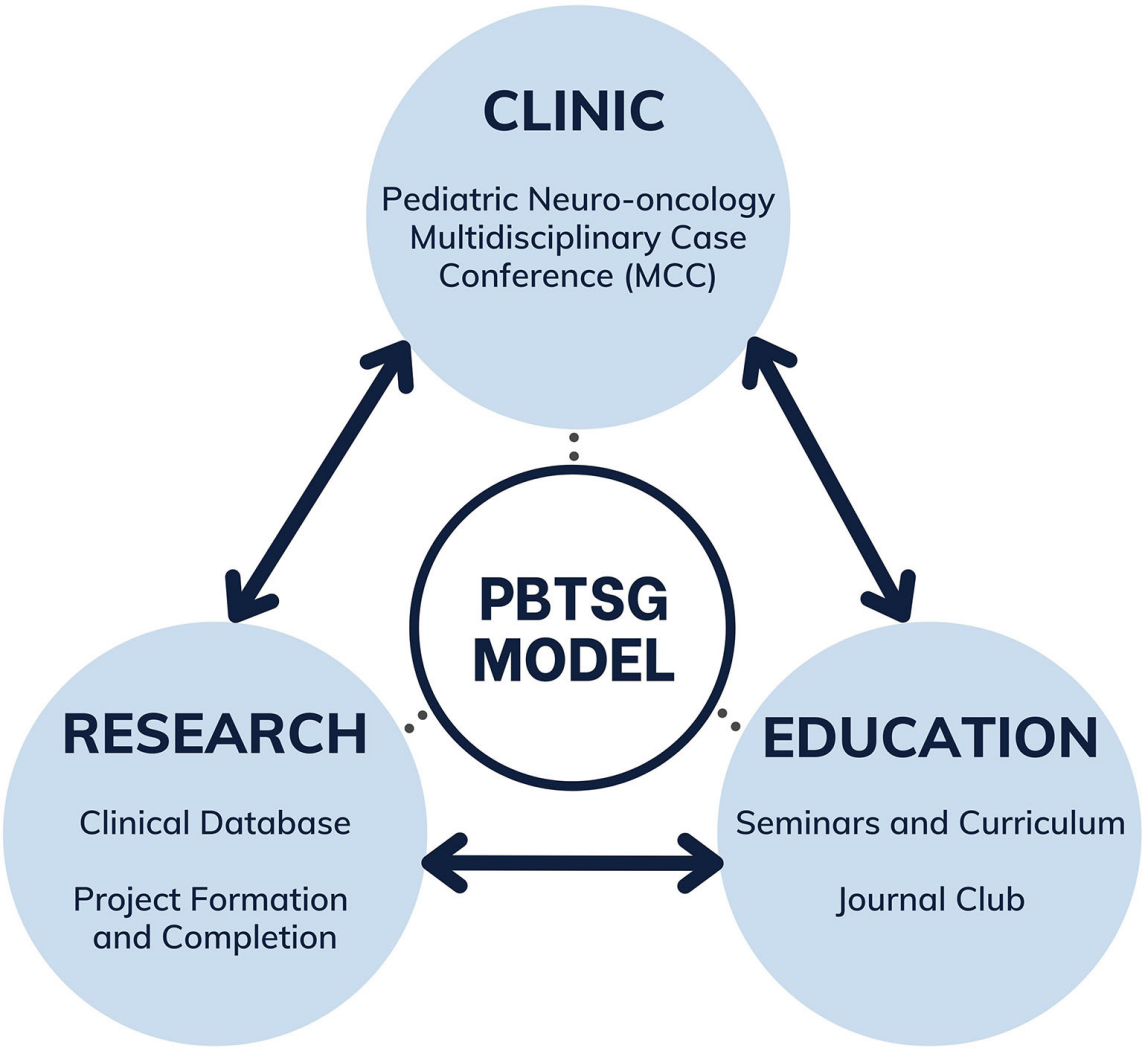
Multifaceted model connecting clinical observations to educational initiatives and research endeavours of the PBTSG.

As members pursued research projects, we acknowledged the need for consistency in data entry and knowledge building (through the weekly MCC). Patient cases discussed during the MCCs are noted for upkeep in our database. These records are then updated by our members at the end of each month. The marriage between the MCC and the database ensures that our data is longitudinally maintained. Moreover, our staff clinicians have started hosting seminars on current topics in neuro-oncology to ensure members are concordant with advancing knowledge in the field. The interconnection between clinic activities, research, and education led to the current model of the PBTSG (**Figure 1**). Recent initiatives have demonstrated increased group communication and productivity from operating on a similar framework (7–9).

### Sustaining Team Productivity

As the database and team membership expanded, a coordinator was appointed for the group and became responsible for administrative tasks, such as assigning data entry to members. While undergraduate student members found time to populate the database by designating PBTSG as an extracurricular activity, their knowledge of neuro-oncology was limited. Moreover, while staff members possessed the most knowledge in neuro-oncology, their protected research time was limited.

**Figure 2:**
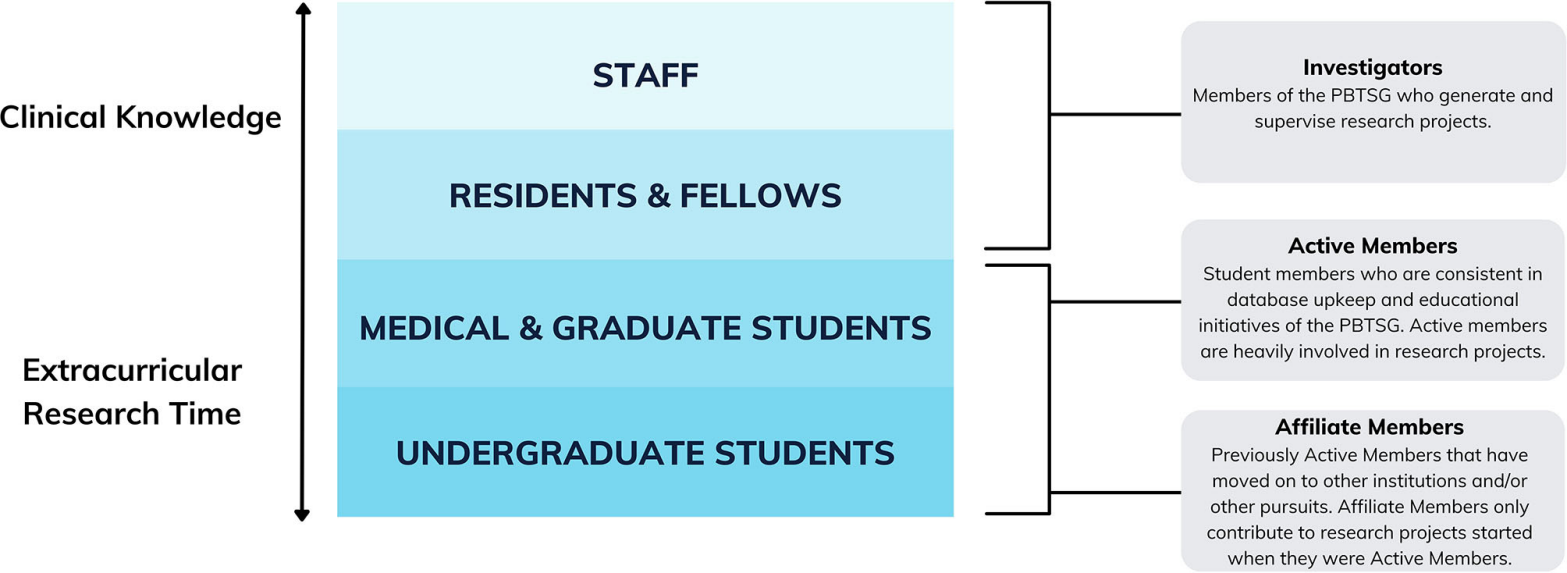
Member tiers of the PBTSG according to clinical knowledge, extracurricular research time, and individual roles.

To resolve this challenge, we implemented a tiered member structure (**Figure 2**). Based on clinical knowledge and research time, this structure represents the multi-generational experiences of our members within clinical neuro-oncology research. Staff clinicians associated with McMaster Children’s Hospital comprise the top of the structure. Through the weekly MCC, staff bridge clinical findings to research potential. Staff also guide student members through research idea development and project supervision, and lead teaching sessions on topics in neuro-oncology to build knowledge within the group. Quality control of the database is also accomplished through staff review.

Residents and fellows participate similarly to staff, as they advise student members on domains such as data extraction and statistical analysis. In addition to project supervision, residents and fellows advise student members on clinical accuracies within the database. This ensures quality control of available data.

Medical and graduate students create and participate in projects while maintaining the database. These members generally have more experience in research, thus providing mentorship to undergraduate students during projects. Meanwhile, undergraduate students maintain the database and also participate in research projects.

The weekly pediatric neuro-oncology MCC sustained productivity by providing student members with real-time exposure to clinical decision making. Members can take their observations and translate them into potential ideas in correspondence with the database and educational curriculum. The weekly conferences also act as a point of contact between staff and student members, allowing them to establish familiarity throughout their time in the group. Furthermore, members are able to observe concepts from our curriculum being applied in a clinical setting and be inspired by the stories of patients.

Ultimately, we optimized the assets of our two cohorts—the invested time and willingness of students to undertake dedicated primary research and the expert knowledge of clinicians—to sustain team productivity.

### Maintaining Communication and Team Dynamics

With the introduction of member tiers, the PBTSG needed to support a large network of staff and student researchers. The tiered structure alone became constrained by gaps in communication between training levels and disciplines. As well, the administrative demands placed upon a single coordinator weakened the team’s communication network. Correspondence between members and the coordinators faltered, leading to disorganization and incomplete projects. As demonstrated by research consortiums in other health disciplines, maintaining robust structure and organization is crucial for research productivity in an interdisciplinary context (10, 11). Moreover, informal transitions within the coordinator role presented difficulty in maintaining group consistency. As each coordinator progressed in their educational journey and departed the team, a dedicated transition period became necessary. Without robust processes to account for these changes, the PBTSG would compromise its sustainability.

To resolve these challenges, a second coordinator was introduced to the group. With double leadership leading to positive outcomes in both business and healthcare settings (12, 13), the dyad leadership model enabled decision-making capable of yielding dividends that exceeded the individual capacity of either coordinator. A second coordinator was also able to offset organizational burdens and conserve institutional memory in the case the other coordinator departed the team. For the member that would fill the departed coordinator’s role, the second coordinator was able to adequately transition them into the role without compromising time.

Team dynamics within the PBTSG were further preserved by creating three member roles: Investigators, Active Members, and Affiliate Members (**Figure 2**). All members convened at monthly general meetings, where Investigators could provide real-time feedback to members’ research projects and educational initiatives.

For Active Members, follow-up protocol was instituted to maintain accountability and address any project concerns in a timely manner. This included monthly progress reports, where each Active Member highlighted their perceived project progress, challenges, and goals every month. Monthly progress reports were then used to guide biannual review meetings, where the coordinators engage with each Active Member on their project progress, future directions, and rapport within the group. This participative governance style of leadership is seen in many productive research collaboratives (14) and should be emulated to ensure long-term success.

#### Group Processes

Every PBTSG member is given autonomy in designing their own research projects. When paired with leadership support, autonomy can be a key driver behind productivity and satisfaction (15). Rather than assigning fixed research topics to each student, students were encouraged to develop organic research questions as they learned how to enter data and populate the database. These ideas were further developed as students made key observations about patterns of data and substantiated their observations with relevant findings in the pediatric brain tumour literature. Altogether, basing projects on members’ specific interests in pediatric neuro-oncology leads to autonomy that can drive efficient project completion and greater quality research output.

Inevitably, challenges arose within the PBTSG, especially during the COVID-19 pandemic. The transition from in-person to completely virtual correspondence during this time made it difficult for members to connect and follow-up with one another. In some cases, this reached a point where studies had stalled, and teams were experiencing significant obstacles to their process. To intervene, the coordinators conducted weekly check-ins with struggling project groups and mutually developed solutions to communication and collaboration concerns. Empathy was also reinforced among members, as groups with greater empathetic communication function better overall (16). For example, we extended project timelines to account for personal changes incurred by the pandemic. We also adjusted data entry schedules to prevent member burnout. It is through maintaining active professional relationships between members that allow each party to achieve their respective goals.

#### Building Community

Hosting regular in-person meetings became difficult due to COVID-19 social distancing guidelines, which led to direct and noticeable impacts on group functioning. Two initiatives were implemented to resolve these challenges. First, a “data entry club” was created to provide Active Members a collaborative avenue for database upkeep. Through these virtual meetings, members are able to interact with one another while maintaining the growth of the database. As well, bimonthly journal clubs were implemented to provide group discussions on current neuro-oncology research. Together, these events allow for interaction on a professional and personal level, and foster a greater sense of community despite the isolation of the pandemic.

## Discussion

### Future Directions

At this juncture, the PBTSG aims to leverage its experiences to maintain a sustainable future. Each year, our coordinators prepare a strategic plan focusing on imminent and long-term goals for education, productivity, and expansion.

As a primary component of our group model, we aim to improve our educational efforts over the coming years. A critical review was undertaken of our curriculum, and our Active Members have since developed a condensed intracranial tumour guide based on the 2016 classification by the World Health Organization (17). This guide eases accessibility to useful tumour information when performing data entry and will be updated to reflect the 2021 classification (18). Another core tenet of the group is self-directed learning, which is reflected in letting students decide their own projects and research avenues. To advance this educational aspect, we have encouraged students to host journal clubs. These will serve to increase student knowledge and engagement, and foster meaningful interactions between student members and staff.

Our second avenue for improvement focuses on increasing group productivity. We aim to strengthen our productivity by diversifying our repertoire of research studies. Our group has primarily been focused on retrospective chart review investigations, which are 1) heavily dependent on sample size to produce meaningful results, and 2) have limited accessibility for newly joined members that have not received access to patient data. In addition to these retrospective studies, we now encourage members to undertake systematic reviews. This will increase our team’s knowledge of research topics, understanding of research methodology, and contributions to the number of systematic reviews in pediatric neuro-oncology.

Our final goal is expansion of the PBTSG. Due to the success of the multidisciplinary neuro-oncology clinic at McMaster Children’s Hospital, we hope to leverage our experiences and assist other institutions in forming groups similar to the PBTSG. Our curriculum, which provides foundational knowledge, can be shaped by new groups according to the knowledge needs of their members. Meanwhile, our model can be used to create a sustainable team. Once PBTSG chapters form at other universities, the opportunity for multicentre research studies could lead to more impactful research at a provincial or national level.

### Recommendations for New Multidisciplinary Research Teams

We have developed three, core recommendations for those wishing to start a similar group to the PBTSG:

#### Develop a Logistical and Team Foundation

Forming a multidisciplinary research team starts with utilizing the activities of its clinic. Recruiting staff, fellows, and residents who collaborate within the clinic is recommended. These individuals will be familiar with its collaborative nature, thus allowing smooth connections between clinic experiences and research endeavours. As well, the diversity of staff membership provides a vast pool of knowledge that student members can learn from.

Logistics such as research ethics board approval and database housing costs should be considered. While these processes vary across institutions, they are universally essential in establishing the basis of a multidisciplinary research team.

#### Use Undergraduate University Students to Drive Group Activities

Students can form a core part of a successful research team by driving group activities and projects. Self-initiative is incredibly important among these members, as it will allow them to progress through projects with minimal supervision. As well, these students should possess strong organizational skills, so that liaison between investigators and members is productive. Faculty members and established members of the group must be willing to teach these students at the onset. This ensures student members can meet expectations and are equipped to handle the commitment required for such a group.

#### Consistently Re-Evaluate Team Activities to Ensure Group Sustainability

Sustainability of a multidisciplinary research team relies upon regular review of the team’s activities. Regular reappraisal of our status, both at the individual level and on an institutional level, is vital for effective resource allocation. Finally, an annual strategic plan and review discussed between the coordinators and principal investigator(s), including both short and long-term goals, will ensure success and growth for posterity. For example, the need to improve communication in the PBTSG resulted in a discussion between the principal investigators and coordinators, which was followed by the implementation of monthly progress reports and biannual performance reviews.

### Limitations

While our recommendations can be generalized to multidisciplinary clinics, it is worth noting that our strategies and outcomes are based on perceived challenges of a single neuro-oncology clinic in a pediatric hospital setting. The experiences of our group are limited to the clinic format, ethics review process, and pool of disciplines available at our institution. Thus, one should consider the unique challenges that may arise when developing the specificities of their own multidisciplinary research team.

## Conclusion

Developing a unique research collaborative from the activities of a longitudinal, multidisciplinary clinic can effectively bridge clinical observations to research endeavours. To sustain a successful team, a logistical foundation, team roles, and communication processes must be established. While our experiences are exclusive to the PBTSG, our strategies may be utilized by multidisciplinary research teams operating across other oncology subspecialties. Furthermore, our framework can be used as the starting point for a standardized framework, as the popularity of multidisciplinary and multi-generational research teams increases.

## Data Availability Statement

The raw data supporting the conclusions of this article will be made available by the authors, without undue reservation.

## Ethics Statement

The authors and research team are registered as principal investigators and co-investigators for project #15-103-C under the Hamilton Integrated Research Ethics Board (HiREB). All information were collected within the limits of the HiREB project approval, and no personal health information or other sensitive data were collected or handled for this manuscript.

## Author Contributions

AG, AF, and SS conceived the project idea. AG, AM, and KW contributed to the design of the project. AG, AM, KW, and MR collected relevant information and data for the project. All authors contributed to the writing, revision, review, and approval of the submitted manuscript.

## Conflict of Interest

The authors declare that the research was conducted in the absence of any commercial or financial relationships that could be construed as a potential conflict of interest.

## Publisher’s Note

All claims expressed in this article are solely those of the authors and do not necessarily represent those of their affiliated organizations, or those of the publisher, the editors and the reviewers. Any product that may be evaluated in this article, or claim that may be made by its manufacturer, is not guaranteed or endorsed by the publisher.
